# Phthisis Pulmonalis—A Clinical Lecture

**Published:** 1885-01

**Authors:** W. D. Bizzell

**Affiliations:** Professor Practice of Medicine; Atlanta, Ga.


### PHTHISIS PULMONALIS.

A CLINICAL LECTURE DELIVERED AT THE SOUTHERN MEDICAL COLLEGE.

BY W. D. BIZZELL, M. D., PROFESSOR PRACTICE OF MEDICINE.

Reported for THE ATLANTA MEDICAL AND SURGICAL JOURNAL.

*Gentlemen:* As I have so often told you, there are two methods of investigation, the rational and the physical; or, rather, the signs of disease can be grouped under two heads, viz: the rational signs or symptoms and the physical signs or symptoms. Sometimes one or sometimes the other furnishes the most conclusive evidence; but the cases are rare in which we do not have to combine both methods of investigation.

The patient, Mr. H., who now stands before you, we propose to interrogate, and if possible come to some definite conclusion respecting the nature of his disease, the extent of its ravages, the local and constitution changes it has effected, and the most hopeful measures at our command for cure or alleviation. First then, as to the rational signs, including age, family history, occupation, personal appearance, previous disease, etc. He says, age about 27, father and mother both dead, mother died of consumption, the cause of death in the father unimportant; of immediate family—one sister and brother living and healthy, one sister dead of phthisis; occupation, house painter.

You will observe as you look at him that he is tall, thin, emaciated, and you will also observe at intervals he coughs and that it is hollow or cavernous. When you inquire as to previous sickness you will learn that while never very stout and subject to frequent attacks of one sort or another, it was not till sometime in January or February of the present year, after exposure to cold and wet in the pursuit of his occupation, that his present sickness takes its origin. Commencing as a cold or bronchial inflammation, it has persisted, and the emaciation and physical appearance which you now behold has been gradually produced.

You learn on inquiry that he has never suffered from hæmoptisis or hemorrhage from the lungs, but you learn that he has suffered much from fevers and night sweats; that the matter expectorated from the lungs is more abundant in the morning, that it consists largely of thick lumps or masses of matter, "solid chunks," as he expressed it, that sink on being placed in water. I also desire to call your attention to another feature in the patient's appearance, and that is the manifest clubbing of his finger tips. This deformity is always characteristic of prolonged suppuration and septicemic fever of a low grade, more especially where the lung is involved, though not absolutely characteristic of phthisis, as I have seen it developed in a very high degree in cases of empyemia of chronic duration.

The subjective and objective answers to our interrogatories have afforded valuable information. The family history is of grave import, for there is nothing better established than the fact that a pre-disposition to phthisis can be transmitted by hereditary descent. The mere fact that one of the parents has suffered from phthisis is of some though not vital importance, but when we learn in addition that the disease has re-appeared in the immediate generation of the patient, and when in addition we are informed that our patient resembles in figure and constitutional peculiarities the phthisical mother, and we are confronted with a history of cough, gradual emaciation, fever, night sweats, heavy purulent expectoration, clubbed finger-tips, there is the strongest presumptive evidence of the presence of phthisis,and probably phthisis well advanced in the third stage. But by physical signs carefully interpreted we will be able to perfect our diagnosis, confidently point out the locality or portion of lung which is being ravaged, whether the first, or stage of deposition, or second, the stage of softening, or the third, or stage of excavation or cavity, or whether in different parts of the lung structure all three stages may not be found. Recollecting, as I have so often told you, that physical signs are indications of physical conditions and not of particular diseases, and must be carefully studied together with all information that can be obtained as bearing on the case, we will now proceed to apply the tests of physical diagnosis to the patient before you. First, we have him divested of clothing down to the waist.

We then learn from inspection: First, a confirmation of the general emaciation; second, you will observe the coming forward of the shoulders; you will also observe the flat and hollowed-out appearance of the upper chest, particularly the sinking in of the upper portion of the chest wall in the sub-clavicular region, causing that bone to assume an unwonted prominence, the ribs seem flattened on either side, as though the thorax had been subjected to strong lateral pressure; in consequence the sternum, particularly the lower half, is arched forward, giving that conformation sometimes called the chicken or pigeon breast on the left side; in particular the cartilages of the lower six or eight ribs bulge forward and the sternum is deflected to the left, the whole making a kind of prominence about the cardiac apex. Though this patient exhibits, in a quite marked degree, this deformity of the chest walls, more marked, perhaps, than is usual, yet it is rare to find a full-rounded, well-developed chest in the subjects of hereditary phthisis, and the more marked the deformity the greater the liability to the subsequent development of the disease. We have no tape-measure, but have no doubt, if tested, expansibility of this patient's chest would be far below what it should be for a person of his height. We now cause him to grasp behind his back the left wrist with his right hand, the hands resting on the lumbar region of the spine; we then instruct him to throw the shoulders back by making tense the trapezius, latissimus dorsi and other muscles involved, by this means the skin and muscles of the anterior chest walls are made tense, and we then proceed to percuss the anterior chest walls after the manner we have already demonstrated. Commencing in the supra-clavicular region, we carefully compare the percussion note on either side; you observe that the percussion note is decidedly dull in both localities, but quite dull on the right side. To show what the normal and healthy percussion note should be, we will now percuss the same region of the chest wall of Mr. S., one of the class, who has kindly afforded us the opportunity of comparison. You will observe the full and healthy development of his chest, and, as I now percuss, you will note the low-pitched, clear note, characteristic of healthy lung structure, a sound you should thoroughly familiarize yourself with by frequent practice on the healthy subject. We now percuss the clavicular and sub-clavicular region of Mr. H. In neither of these localities do we find the percussion note normal; though only slightly changed on the left side, but on the right there is a decided dullness until we approach the lower border of the subclavicular region, when you observe the percussion note changes at about two inches from the outer border of the sternum and on a level with the upper border of the third rib, we get a sound approaching the tympanic in character; we make the patient open his mouth and we get that sound known as the cracked pot or cracked metal, which, if verified in some other investigations we propose to make, will indicate the presence of a cavity communicating with a bronchus.

We now proceed to investigate, by auscultation. As I have already explained to you, this may be mediate or immediate; the ear applied directly to the chest wall, only one thin, soft garment intervening, or the stethoscope can be used. I would advise you to train yourselves in both methods. Not having my stethoscope with me to day, I shall apply my ear directly to the chest, only a soft towel intervening. Unlike the sounds elicited by percussion, you cannot share the sounds with me, and the patient is not strong enough to allow each one of you to come down and examine him for yourselves at this time, so you must be content with such description of the sounds as I will now proceed to give you. I would, however, advise you, two or three at a time, to go over to the Ivy Street Hospital, where he is now being cared for, and familiarize yourself with the morbid sounds which are present. We press the ear down tightly but firmly on the chest, in the apex of the right lung we hear rude respiration, which is higher pitched and more tubular than the normal breezy respiratory murmur. We also note numerous clicks or rales, indicative of small areas of softening and partial obstruction of the minute bronchi. In the left apex the respiratory murmur is feeble and high pitched, and expiration, as compared with inspiration, is somewhat prolonged; or, in other words, we have the sounds that characterize the first stage, or that of the deposition of tubercle. On the right side, at the point in the sub-clavicular region, where we obtained the cracked pot sound on percussion, we encounter that peculiar low-pitched, hollow sound known as the cavernous respiration. The patient speaks, and we get the phenomenon of pectoriliquy; we instruct him to cough, and we immediately get that peculiar succession of sounds which are appropriately called gurgling, from the agitation by the air currents of the liquid which partially fills the cavity which has been produced by softening and disintegration of tubercle and lung structure at this point, showing that the third stage has been reached. Auscultation then informs us, interpreted in the light of our previous investigations, that Mr. H. has tubercles in both lungs; in the left apex the disease is in its incipiency; in the right apex, the disease has reached the second stage; in the lower subclavicular region the third stage. Is there any other test which we could use in the investigation? Yes, we might with our microscope, after the method of Koch, examine the sputum for the peculiar rod-shaped bactrium which he has denominated the bacillus of tuberculosis.

It is now pretty well agreed everywhere that in most cases of tubercle this organism can be found, especially where extensive softening has occurred. Dr. Flint, one of the highest authorities in this country, has carefully investigated the question, and admits that this may be of considerable value in confirming the diagnosis in certain cases. But the evidence is sufficiently complete without having recourse to this expedient.

Having completed the diagnosis, we will allow the patient to retire while we discuss the prognosis, and formulate the treatment. Where the deposit of tubercle is so extensive as in this case, and particularly where we find them already so much disintegrated that the third stage, or conitus is reached, with the attendant emaciation, fevers, night sweats and imperfect digestion, the outlook is gloomy in the extreme; yet, even in cases so desperate as this, much can be done in the way of relief of symptoms, allaying pain and combating fever. To discuss the details of treatment, hygienic or otherwise, would require more time than we can devote to the subject to-day. The night sweats can be readily allayed by giving the patient two or three pellets of extract belladonna at bedtime, or its equivalent of the tincture. The fever, when severe, is best combated by full doses of quinine, as occasion may demand. Cod-liver oil will do good always, provided the patient can digest and assimilate it. Many patients can take a teaspoonful of the remedy who cannot take more. This patient is able to take this small amount and seems benefited thereby. Next to this I prefer the extract of malt in combination with some good wine, with several glasses of cream every day. In all these cases where the febrile reaction is high, there is always evidence of inflammation of lung structure surrounding the softening tubercle, attended with pain, sense of tightness or constriction, a painful and distressing cough and tendency to hæmoptisis. Under these circumstances a good blister will afford great relief, and should never be neglected. The length of time a patient will continue to live under these circumstances, will largely depend upon the care and environment he can command. Much can be done by careful selection of climate. A dry, elevated and stimulating climate, which might have proved beneficial in the early stages, is no longer so suitable. For winter the patient will find certain portions of Florida mild, moderately moist, and comparatively equable, very soothing to his sore and damaged lungs. And if this patient could afford it, I would unhesitatingly recommend his removal there for the winter. From the middle of April to the middle of October, there is no better climate for him than the region surrounding this city. In such a case as this you will understand that the best we can hope for is to prolong and not to cure.

